# The burden of bacteremia and invasive diseases in children aged less than five years with fever in Italy

**DOI:** 10.1186/s13052-015-0189-4

**Published:** 2015-11-20

**Authors:** Chiara Azzari, Maria Moriondo, Pasquale Di Pietro, Cesare Di Bari, Massimo Resti, Francesco Mannelli, Susanna Esposito, Guido Castelli-Gattinara, Antonio Campa, Fernando Maria de Benedictis, Gianni Bona, Lisa Comarella, Katsiaryna Holl, Federico Marchetti

**Affiliations:** Division of Pediatric Immunology, Department of Health Sciences, University of Florence and Anna Meyer Children’s University Hospital, Florence, Italy; Istituto Giannina Gaslini, Ospedale Pediatrico IRCCS, Via Gerolamo Gaslini 5, 16148 Genoa, Italy; Azienda Ospedaliera Universitaria, Policlinico Consorziale di Bari, Piazza Giulio Cesare 11, 70124 Bari, Italy; Anna Meyer Children’s University Hospital, Florence, Italy; Pediatric Highly Intensive Care Unit, Università degli Studi di Milano, Fondazione IRCCS Ca’ Granda Ospedale Maggiore Policlinico, Milan, Italy; Paediatric Division, Bambino Gesù Children Hospital, v. Aurelia Km.30, 00100 Palidoro, Rome, Italy; Ospedale Santobono, Via Fiore Mario 6, 80129 Naples, Italy; Ospedale Gaspare Salesi, via F. Corridoni, 60120 Ancona, Italy; Azienda Ospedaliera Maggiore della Carita’, Corso Mazzini, n. 18, Novara, 28100 Italy; CROS NT, SRL, Via Germania 2, 37136 Verona, Italy; GSK Vaccines, Avenue Fleming 3, 1300 Wavre, Belgium; GSK Vaccines, Via A. Fleming 2, 37135 Verona, Italy

**Keywords:** Fever, Invasive disease, Bacteremia, *Streptococcus pneumoniae*, *Haemophilus influenzae*, Pneumococcal vaccine

## Abstract

**Background:**

Invasive diseases (ID) caused by *Streptococcus pneumoniae* (*S. pneumoniae*), *Haemophilus influenzae* (*H. influenzae*), and *Neisseria meningitidis* are a major public health problem worldwide. Comprehensive data on the burden of bacteremia and ID in Italy, including data based on molecular techniques, are needed.

**Methods:**

We conducted a prospective, multi-centre, hospital-based study (GSK study identifier: 111334) to assess the burden of bacteremia and ID among children less than five years old with a fever of 39 °C or greater. Study participation involved a single medical examination, collection of blood for polymerase chain reaction (PCR) and blood culture, and collection of an oropharyngeal swab for colonization analysis by PCR.

**Results:**

Between May 2008 and June 2009, 4536 patients were screened, 944 were selected and 920 were enrolled in the study. There were 225 clinical diagnoses of ID, 9.8 % (22) of which were bacteremic. A diagnosis of sepsis was made for 38 cases, 5.3 % (2) of which were bacteremic. Among the 629 non-ID diagnoses, 1.6 % (10) were bacteremic. Among the 34 bacteremic cases, the most common diagnoses were community-acquired pneumonia (15/34), pleural effusion (4/34) and meningitis (4/34). *S. pneumoniae* was the most frequently detected bacteria among bacteremic cases (29/34) followed by *H. influenzae (*3/34). Ninety percent (27/30) of bacteremic patients with oropharyngeal swab results were colonized with the studied bacterial pathogens compared to 46.1 % (402/872) of non-bacteremic cases (*p* < 0.001). PCV7 (7-valent pneumococcal conjugate vaccine) vaccination was reported for 55.9 % (19/34) of bacteremic cases. *S. pneumoniae* serotypes were non-vaccine serotypes in children who had been vaccinated. Mean duration of hospitalization was longer for bacteremic cases versus non-bacteremic cases (13.6 versus 5.8 days).

**Conclusions:**

These results confirm that *S. pneumoniae* is one of the pathogens frequently responsible for invasive disease.

## Background

Invasive diseases (ID) are considered any disease where microorganisms are identified in the normally sterile body fluids. Bacteremia may be due to predisposing risk factors (sickle cell anemia, oncologic disease, immunodeficiency, indwelling central catheter, etc.), definitive focal infection (pneumonia, meningitis, cellulites, etc.) or present as occult bacteremia, i.e. the presence of pathogenic bacteria in the blood of a well-appearing febrile child without an identifiable focus of infection. Invasive diseases (ID) caused by *Streptococcus pneumoniae* (*S. pneumoniae*), *Haemophilus influenzae* (*H. influenzae*), and *Neisseria meningitidis* including pneumonia, bacteremia and meningitis and other illnesses, are a major public health problem worldwide. In Europe, estimated annual invasive pneumococcal disease (IPD) incidence per 100,000 population has been reported in hospital-based studies to range from 0.3 to 20.3, and varies by age, with the highest incidence observed in children less than two years of age [1]. Availability of pneumococcal vaccines has led to substantial decreases in overall IPD in the United States and Europe [2–4], and there is also evidence of benefits for the unvaccinated population through herd immunity [5]. Concerns about potential replacement of vaccine serotypes with non-vaccine serotypes still need to be addressed.

Knowledge of Italian epidemiologic features of bacteremia and ID relies on voluntary reporting and sending strains to the national reference laboratory, therefore, the true burden may be underestimated and under-reported [6–8]. More comprehensive surveillance, supported by molecular epidemiological analyses, is considered a priority among all the European countries by the European Centre for Disease Control and Prevention.

We conducted a prospective, multi-centre, hospital-based study to assess the burden of bacteremia and ID, including cost of hospitalization, clinical severity, causative agents and vaccination status, among children less than five years of age with a fever of 39 °C or greater. Additionally, oropharyngeal colonization by the investigated bacteria and the estimated clinical expenses due to bacteremia were examined.

## Methods

### Study objectives and definitions

The primary objective of the study was to assess the incidence of bacteremia and invasive diseases (ID) in children <5 years of age in pediatric hospitals in Italy. Secondary objectives were to determine the clinical severity of bacteremia and describe concomitant contributing clinical factors; to determine the rate of hospitalization due to bacteremia; to identify the causative organisms of bacteremia with a major interest in *S. pneumoniae*, *H. influenzae* and *Neisseria meningitidis* and their susceptibility to antimicrobial agents; to assess the rate of IPD in patients previously vaccinated with pneumococcal vaccine; to assess polymerase chain reaction (PCR) sensitivity in diagnosis of bacteremia; to assess biological samples positive cultures or PCR according to subject’s previous antibacterial treatment; to assess oropharyngeal colonization by the investigated bacteria; to assess the incidence of febrile illness in children <5 years of age; and to estimate clinical management expenses due to bacteremia.

Children were enrolled in this prospective, hospital-based study (GSK study identifier: 111334) at emergency departments or inpatient clinics at participating pediatric hospitals in Genova, Bari, Napoli, Roma, Firenze, Trieste, Ancona, Novara and Milano from May 2008 to June 2009.

### Sample size

The expected number of cases of children with fever of at least 39 °C was estimated based on the hospital activity reports over the previous three years. Sample size was derived from the figures given by each participating centre on the at-risk population (i.e. children <5 years of age with fever ≥39 °C) attending their hospitals in a 12 month timeframe. Assuming compliance to the study requirements (i.e. informed consent of both parents, invasive procedures, etc.) of 33 % from each centre, a mean number of 400 subjects per centre were expected for a total of nearly 4000 enrolled subjects.

### Inclusion and exclusion criteria

Children were eligible if they were less than five years of age, had a fever of at least 39 °C at enrolment or at home with at least one measurement in the 12 h preceding enrolment, and had been a resident in the district of the hospital for at least the last three months. At home, fever may have been measured by parents using any method; at enrolment fever was measured for infants under four weeks using an electronic thermometer in the axilla, and for children four weeks to five years by an electronic thermometer or chemical dot thermometer in the axilla or an infrared tympanic thermometer. Children with a presumptive diagnosis of gastroenteritis or a clinical diagnosis of infectious exantherma were excluded from the study. Informed consent was obtained from each parent/guardian prior to performance of any study-specific procedures.

### Study recruitment and procedures

The study involved a single visit which included a medical examination, collection of a blood sample for local biochemistry and bacteriology (culture), and collection of a blood and oropharyngeal sample for invasive disease or colonization to be sent for PCR to the study laboratory. Data on demographics, medical history, vaccination status and previous antibiotic treatment and current fever treatment were also collected at the visit. A log book with aggregated number of fever and ID cases presenting to the centre, regardless of participation status in the study, was maintained at each study centre.

Bacteremic cases were defined as subjects fulfilling inclusion and exclusion criteria with a blood sample positive for bacteria either by PCR or by blood culture. A case was considered non-bacteremic if the PCR or blood culture testing was negative for the 3 bacteria that were tested. Bacteremia predisposing factors included sickle cell anemia, oncologic disease, immunodeficiency, indwelling central catheter or shunts, agranulocitosis, aplastic anemia, arteritis, renal transplant, congenital heart abnormalities, congestive heart failure, cystic fibrosis, human immunodeficiency virus (HIV), Lyme disease, Kawasaki disease and nephritic syndrome.

Diagnoses of focal infections such as community-acquired pneumonia (CAP), pleural effusion, meningitis, cellulites, lymphadenitis, otitis media, or pharyngitis were made on the basis of available diagnostic findings (such as X-rays or cerebrospinal fluid findings). The same criterion was applied for systemic infections (sepsis). A patient could receive more than one diagnosis. The clinical diagnosis made by the investigator of community-acquired was categorized as either a diagnosis of invasive disease or as a diagnosis of non-invasive disease. Pneumonia, pleural effusion, meningitis and sepsis were considered primary clinical diagnoses suggestive of invasive disease. Although sepsis was considered invasive, it was also examined in a separate category. Other diagnoses such as cellulites, lymphadenitis, otitis media and pharyngitis were considered non-invasive.

### Laboratory analysis

The presence of *S. pneumoniae, Neisseiria meningitidis* or *H. influenzae* type b (Hib) DNA in biological samples and isolates was evaluated by Realtime PCR (RT-PCR) using the *EuSep* screen kit (Eurospital, Trieste, Italy) according to manufacturer instructions. The cycle threshold (C_*T*_) value is the PCR cycle number (out of 45) at which the measured fluorescent signal exceeds a calculated background threshold identifying amplification of the target sequence. If no increase in fluorescent signal is observed after 45 cycles, the sample is assumed to be negative. The presence of *Haemophilus* different from Hib was evaluated by a home-made method, using bexA and ompP2 genes as targets and previously published primers and probes (REfX). The presence of *S. pneumoniae* DNA was confirmed by the amplification of *CpsA* gene in end-point PCR as previously reported [9, 10] and briefly described in the next paragraph. Only samples positive for both *lytA* gene in RT-PCR and *CpsA* in end-point PCR were included in pneumococcal serotyping analysis.

For pneumococcal serotyping, RT-PCR was performed in 25 μL reaction volumes containing 2x *TaqMan* Universal Master Mix (Applied Biosystem, Foster City, CA, USA); primers and JOE labeled probes were used at a concentration of 400 nM; FAM labeled probes at a concentration of 200 nM. Six μl of DNA extract was used for each reaction. All reactions were performed in triplicate. Negative controls (both blood samples from healthy controls and sterile water samples) and positive controls (blood samples or cerebrospinal fluid samples known to be positive for *S. pneumoniae* both by cultural and molecular methods) were included in every run. DNA was amplified in an *ABI* 7500 sequence detection system (Applied Biosystem, Foster City, CA, USA) using, for all the primers couples, the same cycling parameters as follows: 50 °C for two minutes for UNG digestion, 95 °C for 10 min followed by 45 cycles of a two-stage temperature profile of 95 °C for 15 s and 60 °C for one minute. If no increase in fluorescent signal was observed after 45 cycles for any of the primer/probe set, the sample was assumed to be negative with the serotype specific primers and was reported as non-typeable.

For culture purposes, 4–6 ml of blood samples (up to three sets) were taken at the fever peak and immediately sent to the local laboratory; procedures established by each hospital for these tests were used, as described previously [11].

### Health economics

Economic data relevant to expenses due to bacteremia were also collected from a subset of children in order to examine and estimate the associated clinical management expenses.

### Statistical analysis

Demographic and clinical characteristics were tabulated by means of descriptive statistics. Medical history, other relevant clinical information and PCR results were presented by means of frequency and percentage distribution. Descriptive statistics were calculated based on health economic endpoints. Fisher’s Exact test was used for statistical comparison of categorical variables.

Statistical analysis was conducted using *SAS* version 9.2 (SAS Institute, Cary, NC, USA). The study protocol was reviewed and approved by both local and national institutional review boards. The Institutional Review Board (IRB) of the Azienda ospedaliero-universitaria “Maggiore della Carità” in Novara (Principal Investigator: Prof. Gianni Bona) first approved the study, then all the IRBs of the participating centers were notified according to the regulatory laws at the time.

## Results

**Fig. 1:**
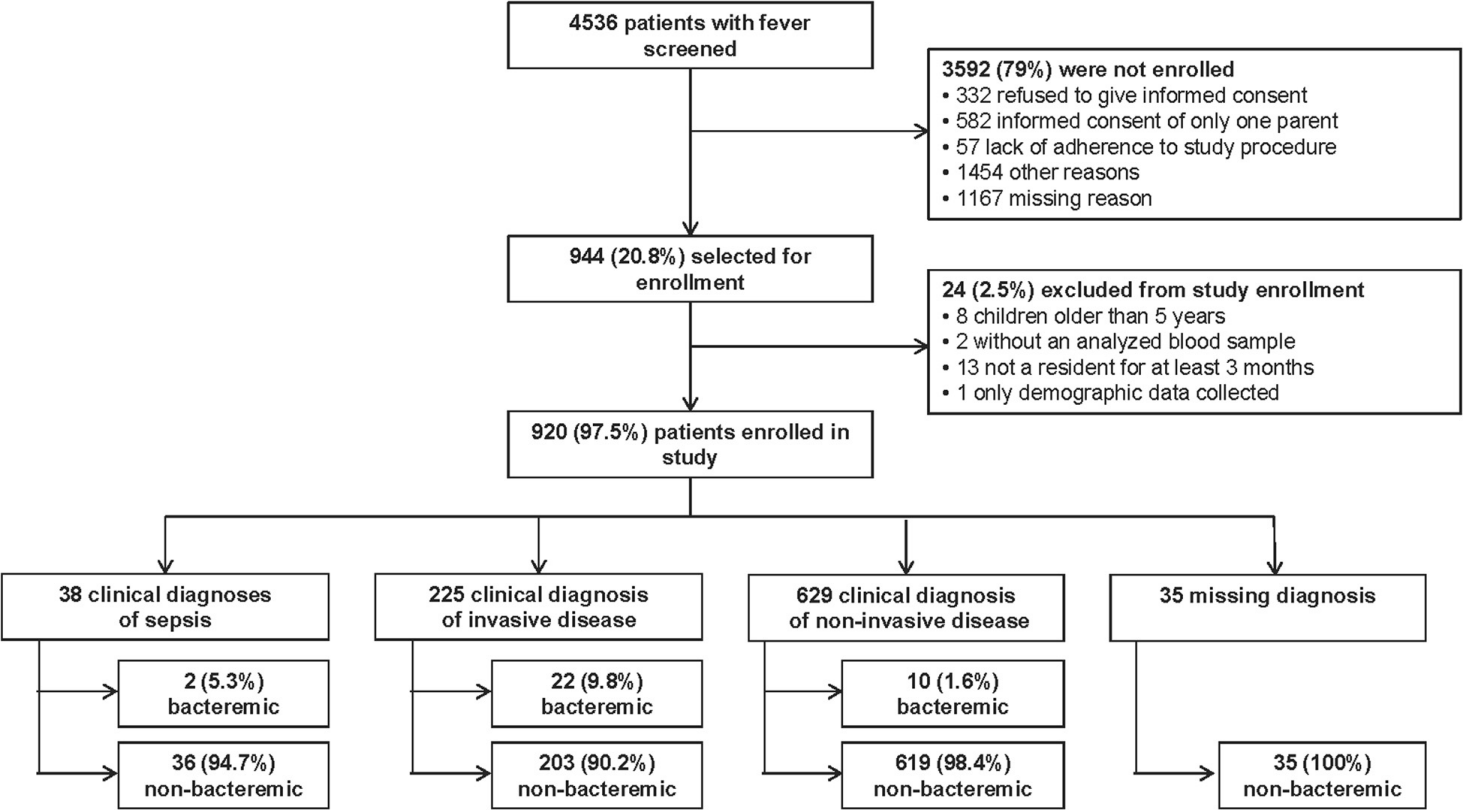
Flow chart for study inclusion and prevalence of invasive disease and bacteremia. Patients could have more than one diagnosis

**Fig. 2:**
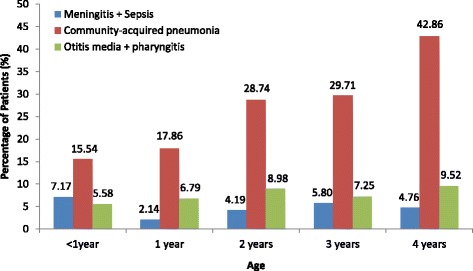
Distribution of confirmed diagnoses by age among the enrolled children

A total of 4536 patients were screened and 944 subjects with high fever were selected for study inclusion between May 2008 and June 2009. The majority of screening failures had a missing reason or ‘other’ reason for non-enrolment (Fig. 1). After exclusion of eight children due to age greater than five years, two due to a lack of an analyzed blood sample, thirteen due to not being a resident for at least three months and one patient for whom only demographic data were collected, 920 children were enrolled in the study. The mean age at enrolment was 23.1 months (range 0–59), and 58 % (529/920) of the children were male. The age group with the greatest proportion of enrolled children was the one year of age group (Table 1, Fig. 2). Among the 814 children with a non-rectal temperature measurement from home, the mean temperature was 39.5 °C; among the 97 children with a rectal temperature home measurement the mean temperature was 39.7 °C. Home temperature was not recorded in nine cases. The mean temperature at enrolment was 38.7 °C (range 34.7-41.1), and 73 % (672/920) of children with fever were hospitalized. At least one predisposing factor was present for 1.4 % (13/920) of children (e.g. oncologic disease, immunodeficiency, intracatheter or central shunt, congenital cardiac anomaly). Thirty-seven percent (339/920) of children reported having received at least one dose of the 7-valent pneumococcal conjugate vaccine (PCV7; *Prevenar*, Pfizer/Wyeth). Antibiotic use before enrolment was reported for 42 % (389/920) of children, and antibiotic use for greater than 24 h was reported for 29 % (262/920) of children. Antipyretic treatment was reported for 88 % (812/920) of children. Systemic inflammatory response syndrome (SIRS) [12] was present in 55 % (507/920) of study patients.

**Table 1:** Characteristics and clinical diagnoses of bacteremic and non-bacteremic cases

	Bacteremic (*N* = 34)	Non-bacteremic (*N* = 886)	All cases (*N* = 920)
Age			
< 1 year	9 (26.5 %)	242 (27.3 %)	251 (27.3 %)
1 year	7 (20.6 %)	273 (30.8 %)	280 (30.4 %)
2 years	8 (23.5 %)	159 (17.9 %)	167 (18.2 %)
3 years	7 (20.6 %)	131 (14.8 %)	138 (15.0 %)
4 years	3 (8.8 %)	81 (9.1 %)	84 (9.1 %)
Prior antibiotic treatment			
No	17 (50.0 %)	481 (54.3 %)	498 (54.1 %)
Yes	16 (47.1 %)	373 (42.1 %)	389 (42.3 %)
Unknown	1 (2.9 %)	32 (3.6 %)	33 (3.6 %)
Prior antipyretic treatment			
No	4 (11.8 %)	79 (8.9 %)	83 (9.0 %)
Yes	30 (88.2 %)	782 (88.3 %)	812 (88.3 %)
Unknown	0	25 (2.8 %)	25 (2.7 %)
Temperature at enrolment (axillary)			
< 39 °C	18 (52.9 %)	452 (51.0 %)	470 (51.1 %)
39–39.4 °C	10 (29.4 %)	239 (27.0 %)	249 (27.1 %)
39.5–39.9 °C	6 (17.6 %)	130 (14.7 %)	136 (14.8 %)
≥ 40 °C	0	63 (7.1 %)	63 (6.8 %)
Missing	0	2 (0.2 %)	2 (0.2 %)
Clinical diagnosis of invasive disease^a^			
Community-acquired pneumonia	15 (44.1 %)	199 (22.5 %)	214 (23.3 %)
Pleural effusion	4 (11.8 %)	4 (0.5 %)	8 (0.9 %)
Meningitis	4 (11.8 %)	2 (0.2 %)	6 (0.7 %)
Sepsis	2 (5.9 %)	36 (4.1 %)	38 (4.1 %)
Clinical diagnosis of non-invasive disease^a^			
Cellulites	1 (2.9 %)	5 (0.6 %)	6 (0.7 %)
Lymphoadenitis	0	12 (1.4 %)	12 (1.3 %)
Otitis media	3 (8.8 %)	38 (4.3 %)	41 (4.5 %)
Pharyngitis	0	27 (3.0 %)	27 (2.9 %)
Genitourinary infection	1 (2.9 %)	45 (5.1 %)	46 (5.0 %)
Other infection	5 (14.7 %)	500 (56.4 %)	505 (54.9 %)

^a^Cases could have more than one primary and/or secondary diagnosis

A total of 225 children had a clinical diagnosis of ID, 38 had a diagnosis of sepsis, and 629 had a clinical diagnosis of non-invasive disease (Fig. 1). Thirty-four of the 920 children (3.7 %) were considered bacteremic cases, having at least one positive sample, detected either by molecular assessment (PCR), culture, or both. Among children with a clinical diagnosis of ID, 9.8 % (22/225) were bacteremic and among children with sepsis, 5.3 % (2/38) were bacteremic, whereas 1.6 % (10/629) of children with a clinical diagnosis of non-invasive disease were bacteremic. PCR was carried out on all the samples, reaching 91 % (31/34) positivity. Culture analysis was performed on 25/34 samples; 36 % (9/25) were positive. Of note, one of the culture-positive samples yielded *Escherichia Coli*, which was not targeted for detection with the PCR. On samples tested simultaneously with both RT-PCR and cultures, 72 % of cases were diagnosed by RT-PCR alone. The overall underestimation factor associated with the use of culture only was 2.53.

The most common diagnosis was CAP, diagnosed in 44.1 % (15/34) of bacteremic cases and 22.5 % (199/886) of non-bacteremic cases (*p* = 0.003) (Table 1). Pleural effusion was diagnosed in 11.8 % (4/34) of bacteremic and 0.5 % (4/886) of non-bacteremic cases (*p* < 0.0001) and in 4/15 of CAP (26.7 %). Meningitis was diagnosed in 11.8 % (4/34) of bacteremic and 0.2 % (2/886) of non-bacteremic children (*p* < 0.0001).

*S. pneumoniae* was the most frequently detected bacteria among bacteremic cases, accounting for 85.3 % (29/34) of cases. *H. influenzae* was detected in three cases (2 non-typeable and 1 capsulated), *Escherichia Coli* in one, and *Neisseria meningitidis* in one (Table 2). The most commonly detected *S. pneumoniae* serotype was 19A, detected in four cases. Among the 15 cases of CAP, 14 were due to *S. pneumoniae,* with three due to serotype 3 and three due to serotype 14. All four cases of pleural effusion were due to *S. pneumoniae,* while three of the four cases of meningitis were due to *S. pneumoniae* with the remaining case was due to *Neisseria meningitidis.* Serotypes are shown in Table 2. Among enrolled cases with oropharyngeal swab results available, 47.6 % (429/902) were colonized by a various mix of the studied bacterial pathogens; 90 % (27/30) of bacteremic cases were colonized compared to 46.1 % (402/872) of non-bacteremic cases (*p* < 0.001).

**Table 2:** Distribution of bacteria and serotypes for the 34 bacteremic cases by clinical diagnosis

Bacterium and serotype	Community-acquired pneumonia (*N* = 15)	Pleural effusion (*N* = 4)	Meningitis (*N* = 4)	Sepsis (*N* = 2)	Cellulites (*N* = 1)	Otitis media (*N* = 3)	Genitourinary infection (*N* = 1)	Other infection (*N* = 5)	Total (*N* = 34)
*Escherichia coli*								1 (2.9 %)	1 (2.9 %)
*Neisseria meningitidis* C			1 (2.9 %)						1 (2.9 %)
*Haemophilus influenzae* (total)	1 (2.9 %)							2 (5.9 %)	3 (8.8 %)
Non-typeable	1 (2.9 %)							1 (2.9 %)	2 (5.9 %)
Serotype missing								1 (2.9 %)	1 (2.9 %)
*Streptococcus pneumoniae* (total)	14 (41.2 %)	4 (11.8 %)	3 (8.8 %)	2 (5.9 %)	1 (2.9 %)	3 (8.8 %)		3 (8.8 %)	29 (85.3 %)
Serotype 1	2 (5.9 %)								2 (5.9 %)
Serotype 3^a^	3 (8.8 %)	1 (2.9 %)							3 (8.8 %)
Serotype 5	1 (2.9 %)	1 (2.9 %)							2 (5.9 %)
Serotype 6A						1 (2.9 %)			1 (2.9 %)
Serotype 7F-7A		1 (2.9 %)	1 (2.9 %)						2 (5.9 %)
Serotype 14	3 (8.8 %)								3 (8.8 %)
Serotype 18	1 (2.9 %)								1 (2.9 %)
Serotype 19A	1 (2.9 %)	1 (2.9 %)		1 (2.9 %)				1 (2.9 %)	4 (11.8 %)
Serotype 19F					1 (2.9 %)	1 (2.9 %)			2 (5.9 %)
Serotype 23F			1 (2.9 %)						1 (2.9 %)
Non-typable	2 (5.9 %)		1 (2.9 %)			1 (2.9 %)		1 (2.9 %)	5 (14.7 %)
Serotype missing	1 (2.9 %)			1 (2.9 %)				1 (2.9 %)	3 (8.8 %)

^a^One patient had community-acquired pneumonia and pleural effusion with *Streptococcus pneumoniae* 3

From parents’ recollection, PCV7 vaccination was reported for 62.1 % (18/29) of bacteremic cases caused by *S. pneumoniae.* Six of the 18 *S. pneumoniae* cases reportedly received three doses of the vaccine (cases were due to serotypes 3, 14, 18, 19A and non-typeable (two)). As for the case due to serotype 18, the laboratory analysis method used could not differentiate serotype 18B (non-vaccine serotype) from 18C (vaccine serotype). Another six reportedly received two doses (cases were due to serotypes 1 (two), 7F-7A, 14, non-typeable and one missing). As for the IPD caused by serotype 14, the two doses had been administered at the fourth and seventh month of life, while the dose that is recommended in the second year of life had not been administered. The remaining six reportedly received one dose (cases were due to serotypes 3, 7F-7A, 19F (two) and non-typeable (two)).

The health economics study included 174 of the enrolled children. The mean duration of hospitalization was 6.47 days (range 1–38) and was longer for bacteremic (13.6 days, standard deviation 9.6) than non-bacteremic (5.8 days, standard deviation 4.0) cases. The mean number of working hours lost was 28.5 (standard deviation 32.4), and was higher for the two bacteremic cases with data available (108, standard deviation 45.3) than for the 75 non-bacteremic cases with data available (26.3, standard deviation 29.6). The mean costs sustained by the family were 107.4 euro (standard deviation 347.6), with a mean cost of 1300 euro (standard deviation 1838) for the two bacteremic cases and 81.2 euro (standard deviation 231.6) for the 91 non-bacteremic cases. As there were only two bacteremic cases with data available on working hours lost and medical costs, comparisons between bacteremic and non-bacteremic cases could not be made.

## Discussion

This study provides much needed information on the burden of bacteremia and ID among children less than five years of age with fever ≥39 °C seeking hospital care in Italy. Thirty-four bacteremic cases out of 920 children with high fever were detected through blood assays; blood analysis (molecular or cultural method) is important for bacteremia diagnosis and should be implemented in children represented by our study population. The proportion of children with bacteremia is likely an underestimation of what is expected in the entire population, since not all patients present at the hospital, and not all presenting patients were enrolled in the study.

CAP and upper respiratory tract infections were the most common infections and reasons for hospitalizations. More than 50 % of enrolled children presented with SIRS (used to measure severity of children seen in hospital), which confirms that this population is clinically relevant. A high proportion of children with fever were hospitalized, however, it should be noted that children presenting at the hospital with fever but with a good clinical profiles would not have been hospitalized and a blood sample would not be drawn, making the child ineligible for the study. Therefore, this figure is likely an overestimation.

*S. pneumoniae* was the most commonly detected (85 %) bacteria under study in bacteremic patients, followed by *H. influenzae* (9 %). Several of the bacteremic cases caused by *S. pneumoniae,* including cases due to PCV7 serotypes, reported having received at least one PCV7 dose. This finding was unexpected, and more detailed information on the timing of vaccination and underlying health of these children are necessary to better understand this finding. Two out of three *H. influenzae* cases were due to non-typeable *H. influenzae,* an emerging pathogen which causes invasive and respiratory infections; a preventive strategy should include as many pneumococcal serotypes as possible and hopefully, in the future, other bacteria causing bacteremia and ID.

Use of the newer molecular techniques for detection of bacteremia is a recent development, and there are very few studies on bacteremia in children in Italy using these techniques [10, 13]. Molecular methods for diagnosing and serotyping bacteremia, such as PCR, do not require viable bacteria, require smaller sample volumes than more traditional blood culture methods, and appear to be more sensitive [10, 11, 13], allowing for a more accurate assessment of the burden of bacteremia. A highly sensitive technique is particularly necessary in the setting of antibiotic use, such as in our study population, where 42 % of children had been exposed to antibiotics. Our results support the higher sensitivity of the molecular techniques versus the blood culture techniques.

Costs generated by a case of bacteremia can be consistent both in the National Healthcare System and in the families’ perspective, with an estimated average direct medical cost of 3306 euro for each hospitalized case (range 511–19,418) based on the estimated daily hospitalization cost of 511 euro [14]. There is a high social cost of more than 100 euro/family on average, and hours lost from work, data which highlight the burden of disease on families of affected children.

A primary limitation of this study was the lower than expected sample size. Despite efforts to take the crowded nature and often overloaded healthcare personnel of the emergency departments into consideration in the study design, the target enrolment was still not met. The study aimed to recruit 4000 patients, but ultimately 920 were enrolled. The limited availability of data on reasons for non-enrolment makes it difficult to determine the primary obstacles to meeting target enrolment.

## Conclusions

*S. pneumoniae* was confirmed to be one of the more frequent pathogen responsible for bacteremia, with over 80 % of cases attributable to the pathogen. The study confirms the higher sensitivity of molecular versus cultural techniques for detecting bacteremia. Overall the study contributes to our understanding of the burden of IPD among Italian children, and provides important data on bacteremia and ID associated with high fever in this population. The study also documents the estimate of the costs associated with bacteremia and sustained by the families. Prevention of bacteremia by means of vaccination is thus warranted.

## Abbreviations

CAP: Community-acquired pneumonia
*H. influenzae*: *Haemophilus influenzae*
Hib: *Haemophilus influenzae* type b
ID: Invasive disease
IPD: Invasive pneumococcal disease
PCR: Polymerase chain reaction
PCV7: 7-valent pneumococcal conjugate vaccine
RT-PCR: Realtime polymerase chain reaction
*S. pneumoniae*: *Streptococcus pneumoniae*
SIRS: Systemic inflammatory response syndrome
